# Processing L2 action verbs shares the same mechanisms for processing L1 items: evidence from a combined behavioral and MEG study

**DOI:** 10.3389/fpsyg.2025.1585897

**Published:** 2025-05-20

**Authors:** Elisa Visani, Davide Rossi Sebastiano, Gioacchino Garofalo, Dunja Duran, Melania Tangorra, Marco Mezzadri, Giovanni Buccino

**Affiliations:** ^1^Fondazione IRCCS Istituto Neurologico Carlo Besta, Milan, Italy; ^2^Università “Vita-Salute” San Raffaele di Milano - Dipartimento di Neuroscienze, Milan, Italy; ^3^Università di Bologna – Dipartimento di Filosofia, Bologna, Italy; ^4^Università di Parma – Dipartimento di Discipline Umanistiche, Sociali e delle Imprese Culturali, Parma, Italy; ^5^IRCCS Istituto Scientifico San Raffaele di Milano, Milan, Italy

**Keywords:** embodiment, embodided cognition, magnetoencephalography, motor system, second language

## Abstract

**Introduction:**

Experimental evidence shows that the sensorimotor system is not only involved in performing actions but also in observing and understanding them, even when verbally described. The involvement of the sensorimotor system in processing action related language material is known as embodiment. Following this approach, language items presented in L1 and L2 should affect motor activity in the same manner.

**Methods:**

This study aimed to investigate the involvement of motor system during the processing of L2 items in a combined behavioral and MEG study. Healthy Italian native speakers performed a semantic decision task on hand and foot actions presented by means of pictures or verbs expressed in English as L2.

**Results:**

Results showed slower hand reaction times and weaker suppression of Beta band power during the processing of hand-related pictures and verbs, as compared to foot-related pictures and verbs, thus suggesting shared neural mechanisms for semantic processing of visually and verbally presented items.

**Discussion:**

This in line with a similar study where Italian verbs were used as language items. However, while no dissimilarity was found in the modulation of the motor system during the processing of verbs presented in L1 and pictures depicting actions in the same category, here, when processing L2 verbs, reaction times were slower than when processing visually presented actions, thus implying an additional cost for processing L2 as compared to L1 verbal items.

**Conclusion:**

We argue that these findings support embodiment, in that they can be explained by a similar, although stronger involvement of the sensorimotor system during the processing of L2 verbal items.

## Introduction

The neural mechanisms involved in processing language material when presented in a second language (L2) are still debated. An influential approach is Ulmann’s differential hypothesis (Ullman, 2001) which claims that L2 acquisition and processing cannot depend on the same brain mechanisms that are used to manage the native language.

Coherent to this approach, in earlier studies in bilingual aphasics, the evidence of selective recovery of one language, but not of both languages, was often interpreted as evidence for a different neural representation of L1 and L2 (Albert and Obler, 1978). This especially when L2 is acquired at a late stage in life (see for a review Indefrey, 2006). More recently, several brain imaging studies have led to the notion that L1 and L2 are processed by the same neural structures (for review see Abutalebi, 2008; Abutalebi and Green, 2016; Perani and Abutalebi, 2005; Sulpizio et al., 2020). However, differential activations were found when the age of acquisition of L2 and the level of fluency are taken into account (Liu and Cao, 2016; Pliatsikas, 2020). Most studies have focused on grammatical and syntactic processing (Dodel et al., 2005; Golestani et al., 2006; Jeong et al., 2007; Rüschemeyer et al., 2005, 2006; Sakai et al., 2004) showing stronger activations in L2 speakers within areas classically known as devoted to syntax (Grodzinsky and Friederici, 2006), including Broca’s region and the adjacent left inferior frontal gyrus, left prefrontal cortex, basal ganglia and cerebellum. These studies included late bilinguals and their findings have been interpreted as due to a stronger effort in processing grammatical and syntax aspects of L2 as compared to L1. There is less empirical evidence for semantics processing of L2 items. However, it is most likely that this stronger effort can be extended also to semantics processing, as revealed in a recent meta-analysis of functional brain imaging studies (Cargnelutti et al., 2019). As compared to L1, processing L2 items led to a greater involvement of cortical and subcortical areas, including those classically considered as related to cognitive functions, possibly recruited to support the language processing (Cargnelutti et al., 2019).

A well-established theoretical framework concerning language processing is known as embodiment. Embodiment maintains that language processing involves the recruitment of the same sensory, motor, and even emotional neural substrates recruited when one executes, perceives or feels the content of language material (Barsalou, 2008; Borghi and Barsalou, 2021; Buccino et al., 2016, 2019; Bucur and Papagno, 2021; Del Maschio et al., 2022; Gallese and Lakoff, 2005; Glenberg, 2010; Jirak et al., 2010; Pulvermüller, 2002; Taylor and Zwaan, 2009).

For example, there is increasing evidence that during action perception the same neural structures necessary for the execution of that action are recruited. Involved neural structures include fronto-parietal areas strictly interconnected anatomically and functionally (for review see Borra and Luppino, 2019). Even more interesting for the present study, the neural structures implicated in the execution and understanding of observed actions also appear involved in the understanding and processing of action related language material (Hauk et al., 2004; Tettamanti et al., 2005; for review see Buccino et al., 2016). Following this approach, L2 language material should be processed in the same sensorimotor and emotional circuits recruited during the processing of L1 language material. This is because a given verbal item, whatever the language used, expresses a specific motor experience. A previous study (Buccino et al., 2017; see also Birba et al., 2020; Kogan et al., 2020) showed that nouns expressing graspable objects in L2 modulated hand motor responses in a similar manner as nouns describing graspable objects presented in L1. Interestingly, similar modulation was also found during the processing of visually presented graspable objects. These findings suggest shared neural mechanisms for processing the semantics of nouns expressing graspable objects and seen objects in the same category, regardless of the presentation modality.

In the present study, we wanted to assess whether seen actions and the processing of verbs presented in L2, expressing actions in the same category, similarly modulate the sensorimotor system. Previous studies (Garofalo et al., 2022; Gough et al., 2012, 2013; Marino et al., 2013, 2014; Visani et al., 2022a, 2022b) had demonstrated that this is the case for both visually presented items (pictures of graspable objects and of hand related actions) and verbal items (nouns of graspable objects or even adjectives expressing their motor properties and hand action verbs) presented in L1. In details, these studies carried out with different techniques have shown that the motor system is recruited quite early, around 150–170 ms, during the processing of verbal items related to concrete actions or graspable objects, respectively. This early involvement leads to slower hand reaction times (RTs) in behavioral tasks. Coherent with these behavioral results, Transcranial Magnetic Stimulation (TMS) studies as well as Magneto-encephalography (MEG) studies showed a decrease of Motor Evoked Potentials (MEPs) and a weaker suppression of Beta band oscillations, respectively. Indeed, this modulation of the motor activity is observed in the same body part typically used to perform the action described by the verb (Buccino et al., 2005; Klepp et al., 2015; Visani et al., 2022a) or to interact with the object described by the noun (Marino et al., 2013, 2014). All these results are interpreted as an interference effect due to competition for neuronal resources (De Vega et al., 2004; de Vega et al., 2013; García et al., 2019; García and Ibáñez, 2016) as the motor system processes the meaning of the action and/or of the object, while simultaneously involved in the requested motor task.

The present study aimed at investigating the modulation of motor responses and of Beta rhythm during the processing of verbs presented in L2, in high competent L2 speakers. Based on the embodied theoretical framework, we expected slower hand motor responses and a weaker suppression of Beta band oscillations during the semantic processing of hand-related verbs and pictures as compared with foot-related verbs and pictures, like those found in studies where verbal items presented in L1 were processed.

## Methods

### Sample size estimation

*A priori* power analysis was conducted using G*Power 3.1 to determine the necessary sample size for an rmANOVA. The effect size, as estimated in a previous study by Visani et al. (2022a, 2022b), was set at a partial eta-squared of 0.46. To control for the risk of Type I and Type II errors, the analysis was set at an alpha level of 0.1 and a power of 0.9, respectively. These parameters were chosen to ensure that the study would have adequate statistical power to detect a meaningful difference between conditions, given the expected effect size. Based on these parameters, the power analysis indicated that a sample size of 16 participants is required to achieve the desired power.

### Participants

Twenty volunteers (14 females, age: 26.2 ± 5.8 years) were recruited for the experiment. All participants were adult (>18 years), right-handed according to the Edinburgh Handedness Inventory (Oldfield, 1971), and they had normal or corrected-to-normal vision. They were native Italian speakers with a high competence in English as L2 (Level C1 of the Common European Framework of Reference for Languages, CEFR). All participants did not show neurological and/or psychiatric disorders and did not assume drugs affecting the central nervous system. The experiment was approved by the local Ethical Committee (Fondazione IRCCS Istituto Neurologico Carlo Besta of Milan, approval number 47/2012) and it was carried out in accordance with the ethical standards laid down in the 1964 Declaration of Helsinki and its later amendments. Participants signed the informed consent before being included in the study.

### Stimuli and task

Stimuli were selected from the English Profile project which is based on data provided by real learners of English, that is what learners throughout the world can do at each level of the CEFR. Since the participants included were all highly proficient (C1) in English, the stimuli used in the study belonged to a range within the B2 level. This type of selection allowed us to present participants with stimuli which could be efficiently processed from a semantic point of view.

From an original list of 30 verbs, 24 were selected to be used in the study after an assessment carried out by 11 native speakers of English who were requested to attribute a score from 1 to 10 to indicate to which extent a verb could be related either to hand or foot. Only verbs with a score of ≥7 for only one of the two effectors were included in the final list (Table 1). Hand- and foot-related verbs were also matched for number of letters (Mean Hand: 4.8; Mean Foot: 5.0 letters; *t*(18.7) = −0.44, *p* = 0.66). Pictures used in the present study are the same used in previous experiments (Visani et al., 2022a, 2022b; Garofalo et al., 2022).

**Table 1:** List of the English verbs.

Foot	Hand
Chase	Draw
Dance	Hold
Flee	Paint
Jump	Rip
Kick	Scratch
March	Sew
Run	Spread
Skate	Squeeze
Slide	Stir
Walk	Unfasten
Wander	Untie
Proceed	Comb

Pictures are available at https://doi.org/10.6084/m9.figshare.17128724.v1.

Pseudo-verbs were built by substituting one consonant and one vowel in two distinct syllables of each verb (e.g., “sprotch”” instead of “scratch”). The scrambled pictures were the actions blurred and/or twisted to the photos depicting both hand- and foot-related actions, so to make them unrecognizable and then meaningless.

**Figure 1:**
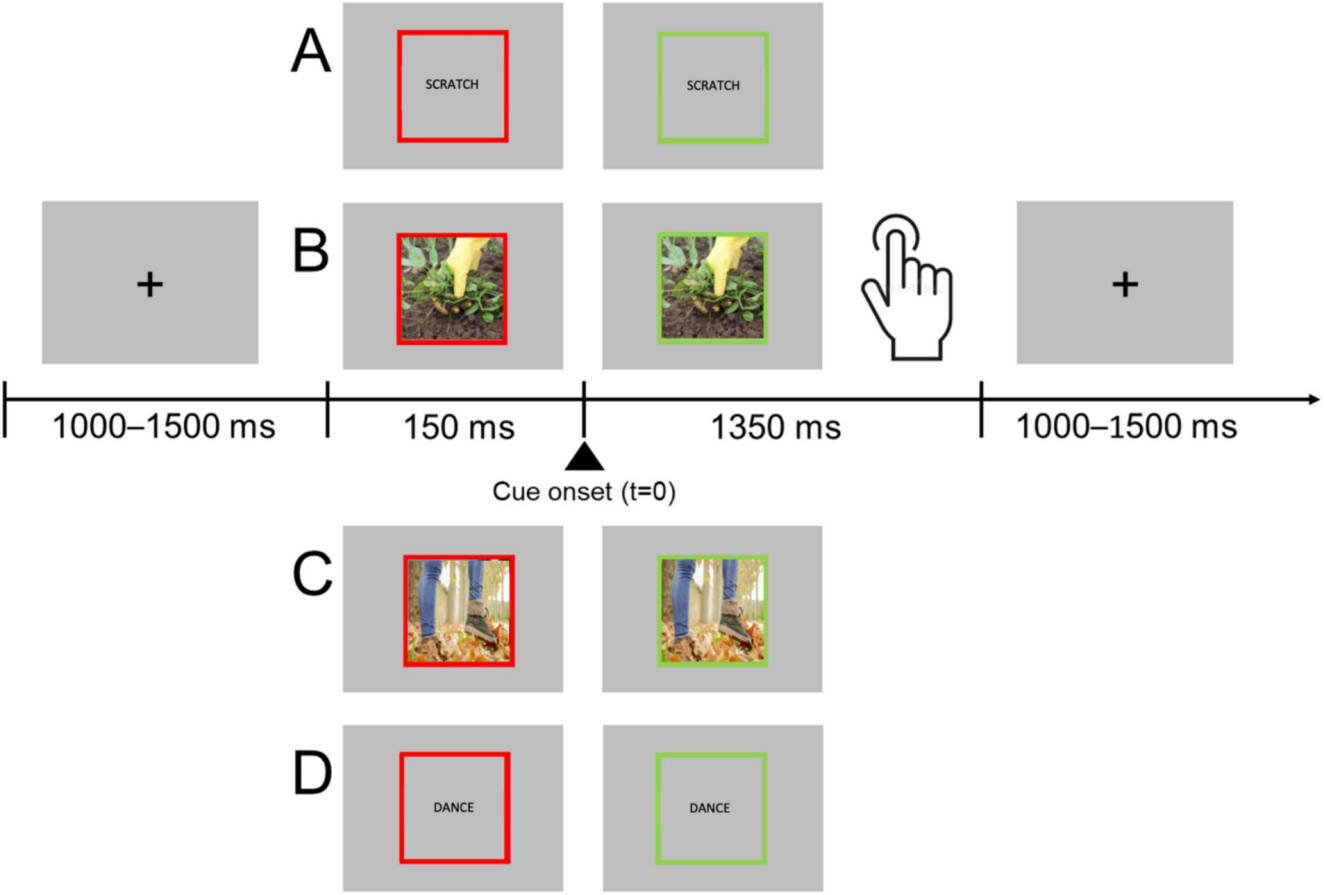
Experimental procedure. Participants were asked to fixate the center of the screen placed in front of them. Each trial started with the presentation of the stimulus surrounded by a red frame. After 150 ms the frame turned green, and the participants were requested to respond. Participants were instructed to respond only if the stimulus was a picture depicting a concrete action (foot or hand action) or a meaningful L2 verb expressing an action in the same categories. The trial ended when participants provided their responses or after 1,350 ms if no response was given. Real stimuli examples: hand-related verb **(A)**, hand-related picture **(B)**, foot-related picture **(C)**, foot-related verb **(D)**. Other stimuli were scrambled hand or foot-related pictures and hand or foot-related pseudo-verbs.

Participants performed a go/no-go task in which they had to respond with a flexion of the right hand to different stimuli, including words (hand- or foot-related verbs) and pictures (hand- or foot- related pictures). They had to refrain from responding when stimuli were pseudo-verbs or scrambled pictures (Figure 1). Participants were told verbal items included verbs. Each trial started with a black fixation cross presented for a random period between 1,000 and 1,500 ms at the center of a gray background. The fixation cross was then replaced by a stimulus item surrounded by a red frame. After 150 ms the frame changed to green, and the participants had to respond or avoid the response. Overall, 96 go stimuli (24 hand-related pictures, 24 foot-related pictures, 24 hand-related verbs, 24 foot-related verbs) and 96 no-go stimuli (24 hand and 24 foot pseudo-verbs, 24 hand and 24 foot scrambled images) were randomly presented in two sessions. Stimuli were delivered using the software package Stim2. Before starting the acquisition, participants underwent a short training session.

### MEG acquisition and pre-processing

A 306-channel whole head MEG system (Triux, MEGIN, Helsinki, Finland) was used to collect the MEG signals. Pairs of electrodes positioned bilaterally 2–3 cm apart over the belly of the right and left flexor and extensor of the wrist were used to simultaneously record surface ElectroMyographic Signals (EMG). Moreover, bipolar electro-oculographic (EOG) and electrocardiographic signals (ECG) were acquired. All signals were sampled at 1 kHz. A 3D digitizer (FASTRAK, Polhemus, Colchester, VT, United States) was used to digitally capture the locations of five coils on the participant’s scalp, three anatomical landmarks (the nasion, right and left pre-auriculars), and additional scalp points before the recording to continuously monitor the participant’s head position inside the MEG helmet and to co-register MEG signals and the template MRI images (see below).

In order to remove external interference and correct for head motions, the raw MEG data were first pre-processed off-line using the spatio-temporal signal-space separation approach (Taulu and Simola, 2006) implemented in the Maxfilter 2.2 (MEGIN, Helsinki, Finland). The data were then band-pass filtered at 0.1–100 Hz. Cardiac and ocular movement artifacts were removed using ICA algorithm based on EEGLAB toolbox (Delorme and Makeig, 2004) implemented in a custom-made MATLAB code (R2021a, Mathworks Inc., Natick, MA, United States), using ECG and EOG as reference. MEG data were divided into epochs ranging from 2 s before to 3 s after the stimulus onset. Epochs showing continuous muscle contraction, identified by visual inspection of the EMG signal, and epochs with sensor jumps were excluded from further analysis. In particular, one epoch was eliminated in two participants. Finally, data epochs were grouped according to the experimental conditions: hand-related pictures, hand-related verbs, foot-related pictures, foot-related verbs. Movement onset was determined by manually tagging the onset of the EMG burst identified as the time point in which the EMG signal exceeded 30% of the maximal voluntary contraction. Reaction times (RTs) were calculated as the interval between the stimulus onset and the movement onset.

### MEG data analysis

All the analyses were performed using Brainstorm software. To generate the realistically shaped single-shell head model, a template brain MRI (MNI/ICBM152, 56), co-registered on MEG data by means of digitized scalp points was used. Dynamic statistical parametric mapping method (Nishitani and Hari, 2000) was employed for the estimation of the brain activity at the source level. The noise covariance matrix was calculated using pre-stimulus baseline period data (−1.5 to −0.5 s).

Individual source maps were spatially smoothed with a Gaussian kernel with Full-Width Half Maximum of 3 mm and were averaged for all conditions. Brain sources were grouped according to Destrieux’s atlas (Destrieux et al., 2010) for further analysis. The precentral gyrus (preCG) was chosen as a Region of Interest (ROI) for the analysis, in line with similar studies (Visani et al., 2022a, 2022b). As defined by the Destrieux atlas, this ROI encompasses the entire motor strip, including cortical representations of the foot, hand, and face. The source time series corresponding to each epoch (−2 to 2.5 s) was extracted from all vertices belonging to the ROI and Principal Component Analysis (PCA) was used to obtain a single time series for each condition for all the successive comparisons (i.e., virtual channel).

Time–frequency representations of virtual channel epochs were computed across frequencies from 1 to 30 Hz (in 1 Hz steps) and time from −2 to 2.5 s (in 0.1 s steps) with a fixed frequency smoothing of 4 Hz by mean of multitapers approach. The relative power change time-course compared to the mean of baseline period (−1.5 to −0.5 s before stimulus onset) was calculated for each epoch and each frequency.

For each participant, the most reactive beta-band frequency was determined in the 13–30 Hz range as the frequency showing the maximal desynchronization value in the period of interest (150–350 ms). Lastly, the values were averaged for each condition separately and, as in the previous study (Visani et al., 2022a, 2022b), the Area under Curve (AuC) in the period of interest was calculated. Analyses were performed by means of custom Matlab code (MATLAB 2021a, MathWorks, Inc., Natick, MA, United States) using functions from the Fieldtrip toolbox (Oostenveld et al., 2011).

### Statistical analysis

To determine if reaction times and beta rhythm AuC were normally distributed, the Shapiro–Wilk test was used.

RTs and AuC of Beta rhythm were separately compared using repeated measures ANOVA (rmANOVA) with Effector (hand, foot) and Stimulus type (pictures, verbs) as within participants factors. The sphericity assumption was evaluated using Mauchley’s test, and the Greenhouse–Geisser degree of freedom correction was applied when appropriate. Where rmANOVA indicates a significant factor or interaction, paired t-tests were applied as *post-hoc* analysis. Pearson correlation coefficient between behavioral data and MEG responses was also calculated. Significance level was set to 0.05 and values are expressed as mean ± standard error of the mean. All statistical analyses were performed using SPSS (SPSS 20, IBM Corp).

## Results

### Reaction times

Four participants were excluded from analysis due to an error rate greater than 10% (error rate ranging from 13.5 to 31.3%; final population: 11 females, 5 males; mean age: 26.6 ± 6.4 years). The overall mean error rate of the remaining participants was 4.9 ± 3.0%. In particular, the rate of commission errors (response to a scrambled picture or pseudo-verb) was 1.6%, and the rate of omission errors (non-response to words and pictures depicting hand or foot actions) was 3.3%. Only correct trials were further analyzed. rmANOVA showed a significant main effect of Effector (*F*(1, 15) = 34.90, *p* < 0.001, η^2^ = 0.69) and a significant interaction Stimulus type X Effector (*F*(1, 15) = 7.39, *p* = 0.016, η^2^ = 0.33). Regardless of the modality of presentation (pictures or verbs), RTs were longer in the case of hand actions as compared to foot actions (pictures: *t*(15) = −3.41, *p* = 0.002, *d* = −0.85; verbs: *t*(15) = −5.96, *p* < 0.001, *d* = −1.5). Moreover, responses to hand-related verbs were slower than responses to hand-related pictures (*t*(15) = −1.85, *p* = 0.042, *d* = −0.46, Table 2).

**Table 2:** Descriptive statistics and Shapiro–Wilk test for Hand RTs.

	Pictures		Verbs		Effector (ms)
	Mean ± SD (ms)	Shapiro–Wilk test (p)	Mean ± SD (ms)	Shapiro–Wilk test (p)	
Foot-related	455.36 ± 89.51	0.062	461.58 ± 75.34	0.193	458.47 ± 81.44
Hand-related	475.60 ± 86.95	0.329	503.94 ± 86.05	0.511	489.77 ± 86.31
Stimulus type	465.48 ± 87.41		482.76 ± 82.42		

### MEG data

With the ICA artifacts rejection procedure, an average of 3.0 ± 0.7 components related to ocular and electrocardiographic artifacts were removed.

**Figure 2:**
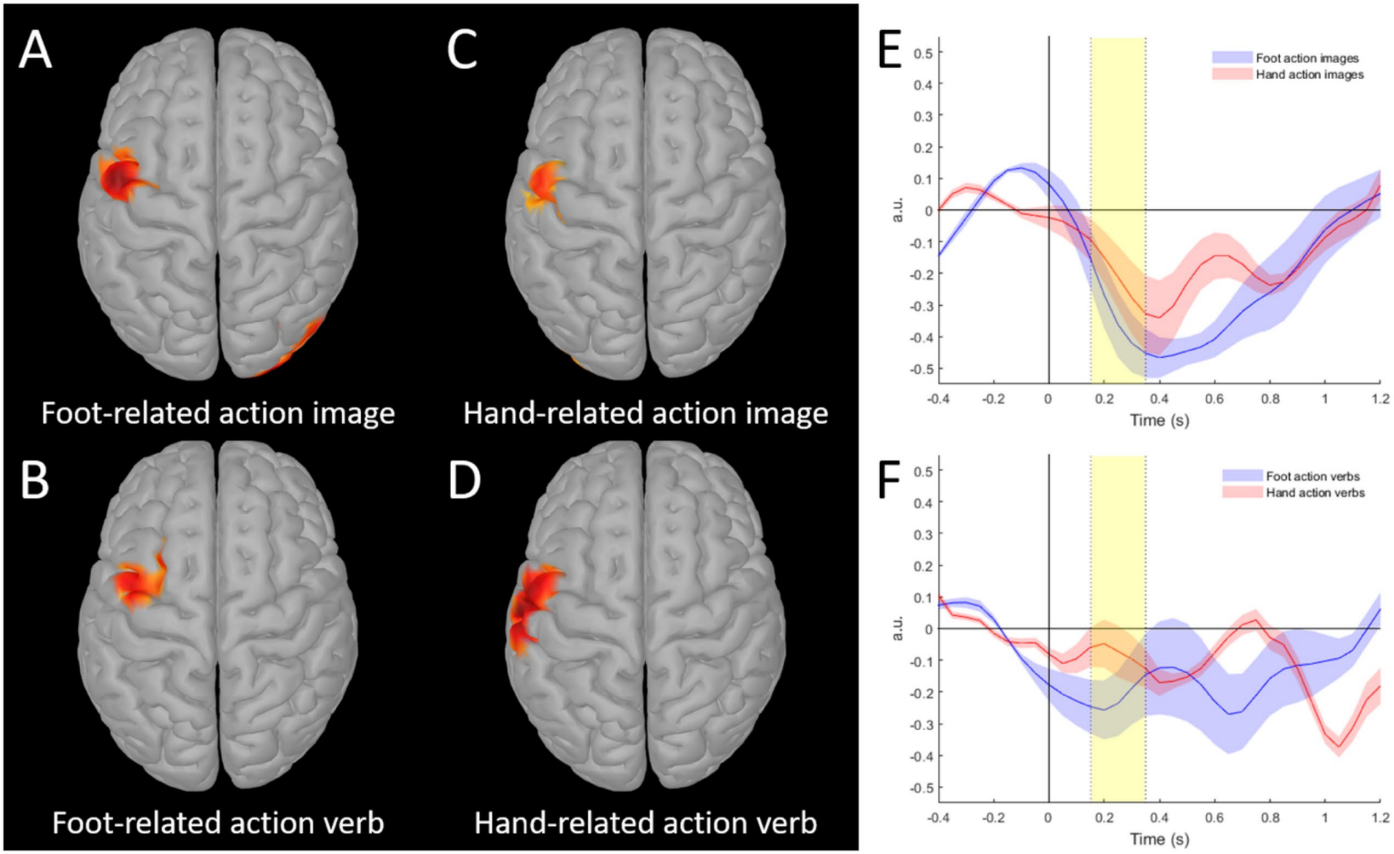
**(A–D)** Grand-average source maps of the response related to different stimuli (**A**: foot-related picture, **B**: foot-related verb, **C**: hand-related picture, **D**: hand-related) obtained with Dynamic statistical parametric mapping in the 150–350 ms period (threshold set to 80% of the maximal activation for each condition). **(E,F)** Beta band modulation of source time series in the selected ROI (precentral gyrus) with respect to pre-stimulus period for images **(E)** and verbs **(F)** stimuli. Highlighted areas indicate the 150–350 ms period; shaded areas indicate standard error of the mean. Dotted lines indicate period for AuC calculation.

rmANOVA showed a main effect of Effector (*F*(1, 15) = 9.813, *p* = 0.007, η^2^ = 0.83). In general, the AuC was greater for the foot-related stimuli as compared to hand-related action ones (Foot-related verbs: −1.41 ± 0.99; Hand-related verbs: −0.59 ± 0.88; Foot-related images: −1.32 ± 0.75; Hand-related images −0.91 ± 0.74). The paired t-tests confirmed the greater AuC in preCG for Foot-related stimuli in comparison to Hand-related stimuli, both visually and verbally presented (Verbs: *t*(15) = −3.10, *p* = 0.007, *d* = −0.78; Pictures: *t*(15) = −1.80, *p* = 0.046, *d* = −0.45) (see Figure 2). No other statistically significant differences were found.

No correlation between behavioral data and MEG responses was found (Foot-related verbs: *r*(16) = −0.134, *p* = 0.621, Hand-related verbs: *r*(16) = −0.116, *p* = 0.670, Foot-related pictures: *r*(16) = 0.240, *p* = 0.370, Hand-related pictures: *r*(16) = 225, *p* = 0.403).

## Discussion

The present study used verbal stimuli referring to hand and foot actions expressed in English as an L2, and pictures depicting actions in the same categories. We collected hand RTs and modulation of Beta rhythm as revealed by MEG during the semantic processing of these stimuli. The findings of the current study revealed a slowing down in hand RTs to hand-related pictures and verbs, compared to pictures and verbs expressing foot-related actions. Additionally, the analysis of MEG signals unveiled the neurophysiological correlates of this effect by showing a modulation of Beta rhythm within the preCG. The Beta rhythm exhibited a milder decrease during the processing of hand-related (both visually and verbally presented) as compared to foot-related ones.

These findings align with the outcomes of earlier behavioral and MEG studies (Garofalo et al., 2022; Visani et al., 2022a) where overlapping visual stimuli and verbal stimuli presented in L1 were used.

The Event-Related Desynchronization (ERD), triggered by Beta rhythm suppression and recorded in the preCG, occurs when motor areas are engaged in executing an action or, to a lesser extent, when individuals observe or mentally re-enact an action (Hari et al., 1998). Our findings are in keeping with previous studies, indicating that this ERD occurs not only during the observation of hand actions, but also during the early processing of hand-related verbs, albeit to a lesser extent (Klepp et al., 2015; Visani et al., 2022a). The presence of ERD for both visually and verbally presented stimuli supports the view that overlapping neural mechanisms and potentially neural structures are active when participants attribute meaning to actions (i.e., semantic processing), regardless of the presentation modality. These results suggest that the brain areas involved in executing actions are also recruited during the semantic processing of those same actions. This highlights the importance of re-enacting motor structures—where actions are represented—in attributing meaning to items that refer to actions.

One might argue that our findings run counter to significant research indicating facilitation of motor activity during action observation (e.g., Cochin, 1999; Fadiga et al., 1995; Nishitani and Hari, 2000; Strafella and Paus, 2000). When actions are conveyed through verbal labels, such as verbs, a proposed dual-stage processing unfolds (Chersi et al., 2010). The initial stage occurs shortly after stimulus presentation (within 200 ms) and appears crucial for comprehension. From a behavioral standpoint, this early processing manifests as a slowing down of motor responses (Boulenger et al., 2006; Buccino et al., 2005; Dalla Volta et al., 2009; de Vega et al., 2013; Marino et al., 2014; Sato et al., 2008). From a neurophysiological perspective, it entails a reduction in Motor Evoked Potentials (MEPs) amplitude, as revealed by TMS (Buccino et al., 2005), and a less pronounced decrease in ERD, as demonstrated by MEG (Klepp et al., 2015; Visani et al., 2022a). The subsequent stage occurs later than 200 ms, after semantic processing has been concluded. Participants, at this stage, exhibit quicker responses, as it happens in the Action-Sentence Compatibility Effect (ACE) (Buccino et al., 2016 for a review; but for a more critical rethinking of ACE also see Morey et al., 2022, Winter et al., 2022) and grasp-compatibility effect (Bub and Masson, 2010, 2012; Ellis and Tucker, 2000; Garofalo et al., 2021, 2023; Santana and De Vega, 2013; Tucker and Ellis, 2004) or display facilitated neurophysiological parameters, when investigated with TMS and MEG (Chersi et al., 2010; de Vega et al., 2013; Fadiga et al., 1995; Klepp et al., 2019; Niccolai et al., 2017; Watkins et al., 2003).

Considering the present results together with those of previous studies indicating a substantial motor equivalence between observed and verbally described actions (Buccino et al., 2016; Garofalo et al., 2022; Hardwick et al., 2018; Marino et al., 2017; Visani et al., 2022a), one might argue that the modulation of motor activity during action observation is similar to that found during the processing of verbally described actions. Specifically, when participants engage in a hand motor response, as in our task, during the semantic processing of a seen action implying the use of the studied effector, there may be a cost at an early stage. After, the semantic processing has been completed then facilitation of action may occur.

What is novel in the present study is that a similar modulation of motor responses and Beta rhythm as revealed by MEG for previous studies in L1 (Klepp et al., 2015; Visani et al., 2022a), also occurs in high competent speakers of English as an L2, thus testifying common shared neurophysiological mechanisms and potentially the recruitment of the same neural structures during semantic processing of verbal items presented in L1 and L2, respectively. In keeping with this, fMRI studies investigating the neural structures involved in processing both L1 and L2 stimuli showed a substantial overlap of cerebral areas in high competent L2 speakers (De Grauwe et al., 2014; Monaco et al., 2023; Tian et al., 2020; Vukovic and Shtyrov, 2014).

The evidence that L1 and L2 modulate in a similar manner behavioral and neurophysiological parameters excludes Ulmann’s differential hypothesis and further supports an embodied perspective of language processing no matter the languages (L1 or L2) stimuli are presented in. It is worth emphasizing that the results found for verbs parallel those found during the early processing of visually presented graspable objects and their corresponding nouns both in L1 and L2 (Buccino et al., 2017; Devereux et al., 2013; Gough et al., 2012, 2013; Marino et al., 2013, 2014; Shinkareva et al., 2011; Visani et al., 2022b).

When considering RTs, there is an important difference between the results of the present experiment and those of a similar one where L1 verbal items were used (Visani et al., 2022a). Namely, while no dissimilarity was found in the modulation of the motor system during the processing of verbs presented in L1 and pictures depicting actions in the same category, the present findings show that, when processing L2 verbs, RTs were slower than when processing visually presented actions, thus implying an additional cost for processing L2 verbal items as compared to L1 items. Our findings are in line with results obtained in other studies (Boos et al., 2022; Hut and Leminen, 2017). In details, Hut and Leminen, and Boos et al. found that there is a cost when processing language items presented in L2 as compared to L1. When two native languages, as in the study by Hut and Leminen are considered, this difference is not present.

In a hard embodied view of language processing (Buccino et al., 2016), language is grounded in sensorimotor and emotional experience. Hence, understanding the content of any verbal item implies the reenactment of the neural structures where those experiences are coded. Embodiment foresees that L2 and L1 verbal labels share the same neural representations. Hence it may be that while processing L2 verbal items participants also re-enacted the correspondent L1 verbal labels, since the semantics of both items relies on the same motor experience. This strategy, in turn, might have led to an additional time for processing L2 items, as revealed by an additional increase of reaction times at behavioral level, despite the lack of this modulation on ERDs. At behavioral level, similar results were found when comparing RTs recorded during processing L2 nouns expressing graspable objects and pictures depicting objects in the same category (Buccino et al., 2017).

An alternative but not mutually exclusive explanation for the present results could be that during language acquisition processes, different early sensorimotor and emotional experiences are labeled with words belonging to the L1 or potentially to the languages the child is exposed to from birth or very early in life. For example, when an anglophone child acquires the ability of walking, this motor experience is labeled with the correspondent English word. When learning a second language later in life, even in high-competent speakers, as those recruited for the present study, the word presented in L2 is disfacilitated in comparison with the correspondent L1 word. This in turn will cause a further slowing down of motor responses, as the present results for verbs, as well as the ones for nouns (Buccino et al., 2017) seem to prove. In keeping with this speculative idea, Hut and Leminen (2017) showed that there is no cost when switching from two L1s, while this occurs when switching from a later learned L2 to either L1s.

Summing up, the present findings are well explained by the embodied theoretical framework and taken together with all the others quoted above, support the adaptive control hypothesis (Green and Abutalebi, 2013) which assumes the presence of a controlling system in the brain responsible for switching from one language to the other, thus envisaging that there is a cost when using L2 in a single language context, as the one presented in our experiment. In fact, in this case, L2 is in a competitive relationship with L1. This even more when one assumes that processing L1 and L2 items share common neural substrates and mechanisms.

In our view, the present findings may have a role in language education areas. Since experience seems to be the core of any language acquisition processing and languages are grounded in the neural structures coding for sensorimotor and emotional experiences, it may be suggested that promoting experiential learning approaches (Buccino and Mezzadri, 2015; Macedonia, 2014) is relevant also during the language teaching process. In fact, pivotal studies show that when learning vocabulary in L2 both high and low performers benefit from sensorimotor learning (Macedonia and Repetto, 2016). In a similar way, Andrä et al. (2020) demonstrated that learning foreign language words with pictures and gestures is helpful for learners, because pictures and gestures allow both kids and adults to experience the meanings of words through multiple senses.

## Data Availability

The raw data supporting the conclusions of this article will be made available by the authors, without undue reservation.
